# Soft-bound Synaptic Plasticity Increases Storage Capacity

**DOI:** 10.1371/journal.pcbi.1002836

**Published:** 2012-12-20

**Authors:** Mark C. W. van Rossum, Maria Shippi, Adam B. Barrett

**Affiliations:** Institute for Adaptive and Neural Computation, School of Informatics, University of Edinburgh, Edinburgh, United Kingdom; Gatsby Computational Neuroscience Unit, University College London, United Kingdom

## Abstract

Accurate models of synaptic plasticity are essential to understand the adaptive properties of the nervous system and for realistic models of learning and memory. Experiments have shown that synaptic plasticity depends not only on pre- and post-synaptic activity patterns, but also on the strength of the connection itself. Namely, weaker synapses are more easily strengthened than already strong ones. This so called soft-bound plasticity automatically constrains the synaptic strengths. It is known that this has important consequences for the dynamics of plasticity and the synaptic weight distribution, but its impact on information storage is unknown. In this modeling study we introduce an information theoretic framework to analyse memory storage in an online learning setting. We show that soft-bound plasticity increases a variety of performance criteria by about 18% over hard-bound plasticity, and likely maximizes the storage capacity of synapses.

## Author Summary

It is generally believed that our memories are stored in the synaptic connections between neurons. Numerous experimental studies have therefore examined when and how the synaptic connections change. In parallel, many computational studies have examined the properties of memory and synaptic plasticity, aiming to better understand human memory and allow for neural network models of the brain. However, the plasticity rules used in most studies are highly simplified and do not take into account the rich behaviour found in experiments. For instance, it has been observed in experiments that it is hard to make strong synapses even stronger. Here we show that this saturation of plasticity enhances the number of memories that can be stored and introduce a general framework to calculate information storage in online learning paradigms.

## Introduction

Long term synaptic plasticity has been established as one of the most important components for learning and memory. In parallel with experimental investigations, numerous computational models of synaptic plasticity have been developed to simulate network function and to establish the fundamental characteristics and limitations of plasticity. Despite the complexity of the underlying neurobiology, theoretical studies have in the interest of tractability mostly focused on highly simplified plasticity rules [1]. However, more realistic models are now becoming possible in the light of more detailed experimental characterization of synaptic plasticity [2], [3].

One such experimental finding is that strong synapses are harder to potentiate than weak ones, that is, the percentage increase in strength is significantly smaller for strong synapses than for weak synapses [4], [5]. Meanwhile, synaptic depression protocols lead to a percentage decrease in strength independent of strength itself [6]. This phenomenon has been observed under both classical and spike timing dependent plasticity protocols [7], and is known as soft-bound or weight-dependent plasticity (see Discussion for possible biophysical correlates). Soft-bound plasticity contributes to saturation of LTP when one tries to induce it repeatedly. Observation of LTP saturation has been used as evidence that synaptic plasticity did occur during some earlier learning protocol [8], [9].

Soft-bound plasticity automatically constrains the synaptic weights, and thereby resolves simply, but effectively, the danger of unconstrained plasticity, namely that on repeated activation, synaptic strength would grow indefinitely. In many modeling studies weight dependence is ignored, instead hard-bounds are typically introduced that cap the minimal and maximal synaptic weights (also known as weight clipping), which are often supplemented with constraints on the total weight [10], [11]. In other plasticity rules, such as Oja's rule [12], weight dependence might be present but it is not biologically motivated. However, including weight dependence in plasticity rules is not just a minor fix noticeable only if synapses reach extreme values. It has profound consequences for plasticity and its dynamics: First, it leads to unimodal synaptic weight distributions [13], [14], consistent with distributions observed both in electro-physiological [15] and in spine size data [16]. Second, it weakens competition between synaptic inputs [17], [18] and instead causes the synaptic weight to depend smoothly on the correlation between inputs [19], consistent with recent data [20]. Finally, as a result of the weaker competition, for identically sized synaptic updates soft-bound plasticity is less stable compared to hard-bound plasticity [21].

Despite the experimental evidence for soft-bound plasticity rules, the effect of weight dependence on information storage is not well understood [22]. A priori it is not clear whether soft-bound plasticity is better or worse for information storage compared to hard-bound plasticity. In the case of *discrete* synapses it has been suggested that soft-bounds fundamentally limit memory lifetime [23]. Analysis of soft-bound plasticity is complicated by the fact that when plasticity depends on the synaptic weight, it will depend on the history of the synapse. Here we study a plasticity process that is continually on-going and which has started a long time ago, so that the distribution of synaptic weights has reached an equilibrium. We introduce an information measure for such on-line learning schemes. We show that soft-bound plasticity leads to a 18% higher information capacity and find strong evidence that soft-bound plasticity optimizes storage capacity. Moreover, the memory lifetime of soft-bound plasticity is longer than for hard-bound plasticity. Thus, soft-bound plasticity not only helps to constrain plasticity, but it also increases capacity.

## Results

**Figure 1:**
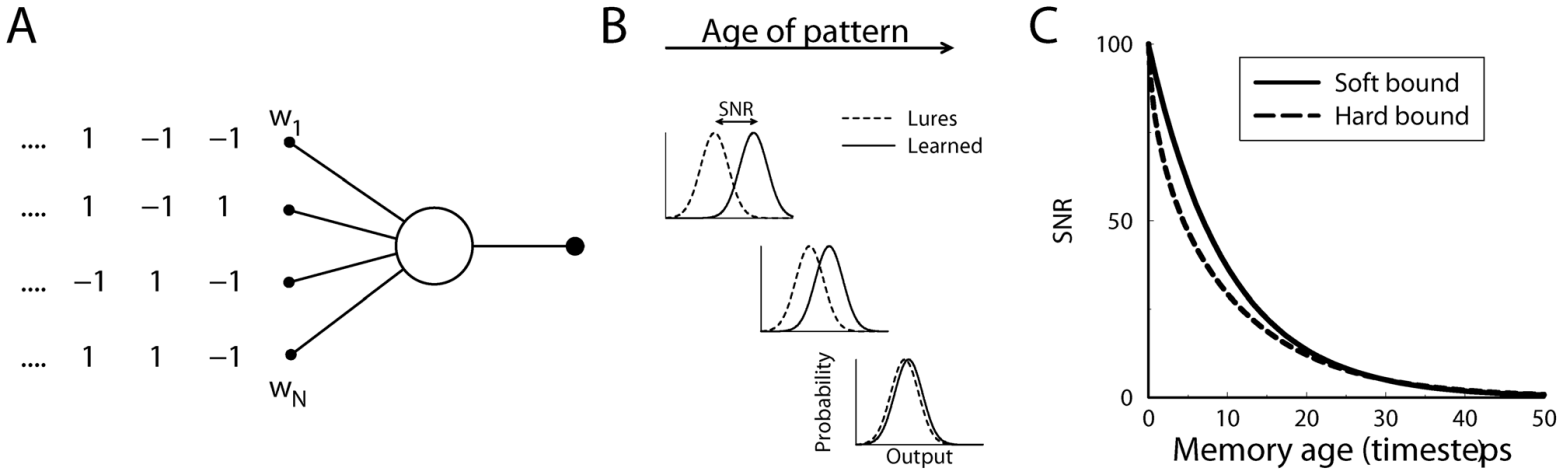
Diagram of the single neuron recognition task. A) A neuron receives binary pattern inputs. At each time-step a new pattern is presented and the weights are updated according to the input value. The neuron's output equals the weighted sum of the inputs. B) The neuron has to remember the presented patterns. When tested, learned patterns lead to a larger output (solid curve) than lures (dashed curve). As the memory of the pattern ages and is overwritten by new patterns, the output of the neuron in response to the pattern becomes less distinct and the signal-to-noise ratio decays. The performance is measured by the signal-to-noise ratio, a measure of the distance between the two output distributions. C) The decay of the signal-to-noise ratio for soft-bound and hard-bound plasticity rules as a function of the age of the pattern. The synaptic updates were set so that both rules led to an initial SNR of 100 right after the pattern was presented (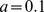, 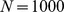). For both plasticity rules the SNR decays, but it decays slower for soft-bound plasticity.

To understand how different plasticity rules determine information capacity, we consider a simple, single neuron learning paradigm. The setup is shown in Fig. 1A. The neuron receives 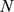 synaptic inputs. Each input has a plastic synaptic weight 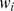. Every time-step we present a different synaptic input pattern. The pattern's elements 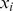, are uncorrelated binary variables, with +1 and −1 occurring with 50% probability (see below for variations). The neuron's output equals the weighted sum of the inputs, 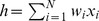. The plasticity rule depresses synapse 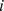 with an amount 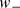 (with 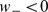) when its input is low, so that 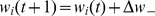,, and a high input potentiates the synapse, 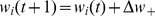. These updates are independent of post-synaptic activity (but see below). As a result the next time the same pattern is encountered the response of the neuron will be higher.

The task of the neuron is to recognize the patterns that it has encountered previously. To measure the performance we periodically interrupt the learning and test the neuron with both previously presented patterns (labeled 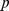) and lures (labeled 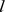) that were not presented before. Based on the output it has to be decided whether the pattern has been seen before or not. This very simplest of tasks can straightforwardly be extended to a supervised associative learning scheme in which some patterns are associated to a high output and others to a low output. Hereto patterns that should give a high output follow the above scheme, while patterns that should give a low output, potentiate synapses with low inputs and depress synapses with high inputs [24], [25].

The model is agnostic about the precise timescale and brain area involved - cortex and hippocampus come to mind. It is also possible to adopt a variant in which only a fraction of all presented patterns is learned. For instance, the synaptic plasticity might only occur with a certain probability, or plasticity might occur only if some additional signal (for instance signalling reward or relevance), lifts the postsynaptic activity above a certain plasticity threshold. Either mechanism would slow down the learning and forgetting equally, but would not otherwise change our analysis [26], [27].

Because the neuron sums many inputs, the output distribution is well approximated with a Gaussian distribution. This holds independently of the *weight* distribution (by the law of large numbers, even with a uniform weight distribution the output distribution will still tend to a Gaussian). Indeed, simulations with and without this Gaussian assumption gave virtually identical results. Using the Gaussian approximation, a signal to noise ratio (SNR) can be used to characterize the difference in the response between patterns and lures. With 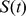 we denote the SNR of a pattern presented 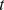 time-steps ago, Fig. 1B,
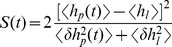
(1)where 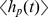 and 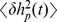 denote the mean and the variance of the output in response to a pattern learned 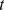 time-steps ago; 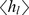 and 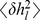 denote the mean and variance of the output in response to a lure.

In many storage capacity studies weights are initialized to zero, the number of items to be learned is fixed and learning stops after all the items have been presented, or once the task as been learned [1], [28], [29]. In such schemes the memory for each item is typically equally strong. Thus a single number characterizes the performance, and plasticity rules can be designed to optimize it [30], [31]. In contrast, we consider an on-line learning scheme. In on-line learning the plasticity never stops, which is arguably more relevant biologically. Old patterns are continuously forgotten as new ones are learned, known as the ‘palimpsest’ property [32]. The definition of memory capacity requires more care for on-line learning. The quality of a memory, expressed by the signal-to-noise ratio of the neuron's output, decays with age of the pattern. Fig. 1B shows the probability distribution of the output of the neuron. As the memory of the pattern ages and gets overwritten, the output of the neuron becomes indistinguishable from the response to a lure, Fig. 1C.

### Soft-bound versus hard-bound plasticity

The central question we address is which plasticity rules lead to the best performance. We focus on the effect of the weight dependence and restrict ourselves to plasticity rules that are local (only dependent on the pre- and post-synaptic activity at that synapse) and incremental (have no access to the patterns presented earlier). We implemented first two plasticity rules, a hard-bound and a soft-bound one.

For the hard-bound plasticity rule, potentiation occurs when the input 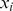 is high and when the input is low, the synapse depresses: For the hard-bound rule we use
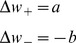
(2)For these plasticity rules hard-bounds on the synaptic weight need to be imposed to prevent unlimited growth; these were set at 0 and +1. The results do not depend on the choice of boundaries, as long as there is feedforward inhibition tuned to the mean weight (see below). The magnitude of 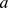 and 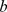 determines how much the weight is updated per potentiation or depression event. We balance potentiation and depression, i.e. we set 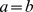, which is optimal in our scenario (see below for unbalanced parameters). In this case, the weight distribution is uniform. We can include dependence of the plasticity on the level of post-synaptic activity, which, dependent on parameters, can lead to a bi-modal weight distribution, as in STDP [13]. This bi-modality is weak as the inputs are uncorrelated in our setup. Performance is decreased when such dependence on post-synaptic activity is included (not shown).

Secondly, we implement a soft-bound plasticity rule. Here the absolute amount of potentiation is weight independent, while the depression is proportional to the weight,
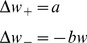
(3)This mimics experimental data [4], [6], [7]. Note that in experimental studies the *relative* amount of plasticity is typically reported. It was found that relative amount of depression is approximately constant (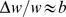) and potentiation is inversely proportional to weight (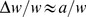). This leads to the above plasticity rule for the absolute amounts. No bounds need to be imposed with the soft-bound rule, the plasticity is intrinsically bounded. For small updates (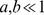) the soft-bound plasticity yields a Gaussian weight distribution, centered around a mean weight 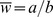, and a variance 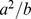.

The decay of the SNR for both hard- and soft-bound plasticity rules is shown in Fig. 1C. Here the plasticity parameters were set such that the signal-to-noise at time 0, i.e. the initial strength of the memory tested immediately after presentation, was 100 in both cases. For soft-bound plasticity the decay is exactly exponential, 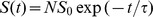, where the time-constant 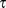 is the memory's decay time, and 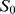 is the initial memory strength per synapse and 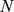 is the number of synapses. For hard-bound plasticity rules, the decay is not exactly exponential (see Models for the exact expression), although an exponential fit can still be used to characterize performance. Importantly, the soft-bound plasticity decays more slowly and thus retains the memory longer.

Ideally one has a slow forgetting and a strong initial memory, however this is impossible to achieve. High plasticity rates (large 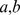) lead to a strong memory of recent patterns (large 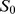) but also rapid forgetting (short 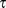), as old memories are overwritten by new ones. On the contrary, small plasticity rates will extend the memory time but will also lead to a reduced strength of the memory. Thus, in online learning, these two competing quantities characterize the memory performance and there is a trade-off between them. Here we will use two different approaches to solve this trade-off and express memory capacity as a single number, thus enabling quantitative comparison between hard-bound and soft-bound plasticity. First, we use information theory to calculate the information per synapse and, second, we use a more traditional signal-to-noise argument to calculate the memory lifetime.

### Information theory

The first way to resolve the trade-off uses mutual information. The mutual information expresses how many bits of information are gained about the novelty of a pattern by inspecting the output of the neuron when it is tested by lures and learned patterns. We pass the output of the neuron through a threshold with value 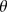. This thresholded response, 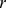, equals zero when the summed input is less than the threshold 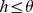, and 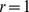 when 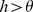. The mutual information between the response and the pattern is given by
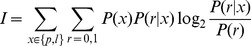
(4)where 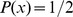 is the probability for a pattern of either class: previously presented (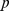) or lure (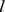). 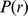 is the probability for a certain response, and 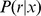 is the conditional probability for a given response on a given pattern class. Concretely, the expression contains the probabilities that a learned pattern is correctly recognized 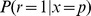, that a lure pattern is correctly identified 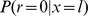 and the two error probabilities 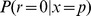 and 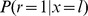. To obtain the total information stored per synapse, we sum the information over all presented patterns and normalize it by the number of synapses. We call this the information per synapse, termed 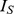. Thus, when a neuron with 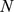 synapses would be able to perfectly recognize 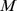 patterns, the information per synapse would be 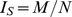.

**Figure 2:**
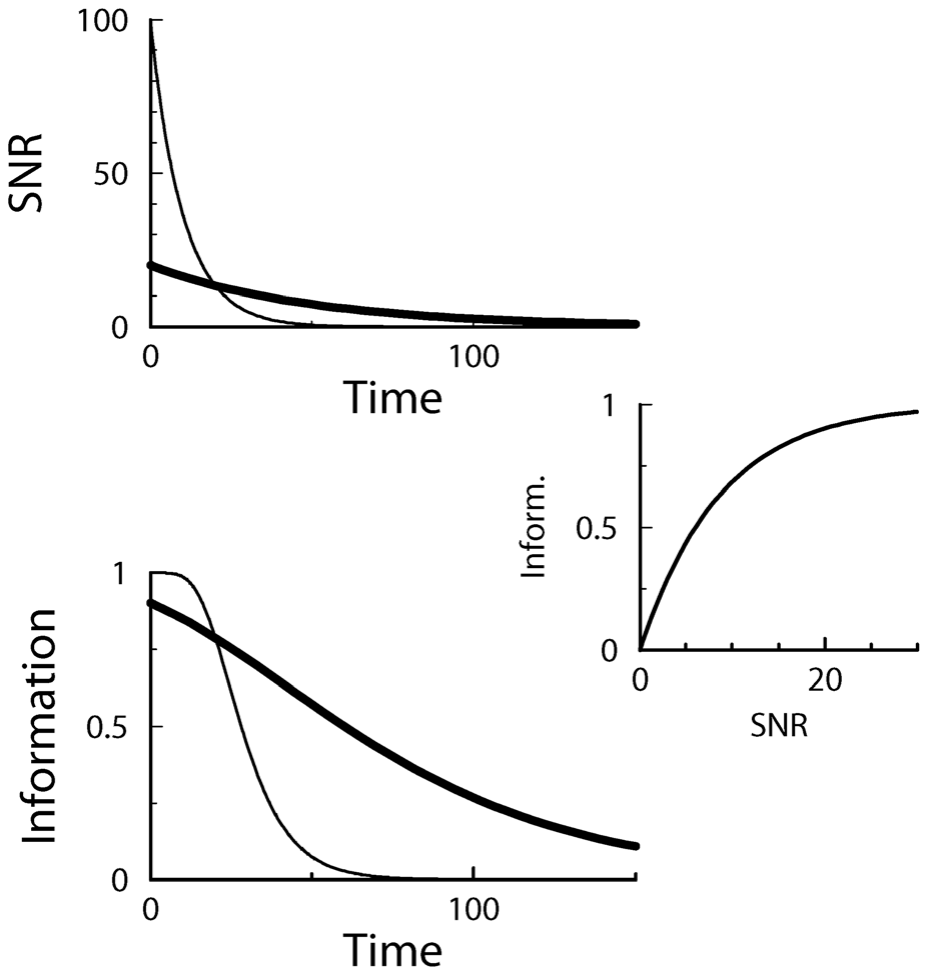
Relation between the information and the SNR. Top: The SNR decay curves versus pattern age for soft-bound plasticity with a large synaptic update (thin curve), and soft-bound plasticity with a small update (thick curve). Although the rules trade off between slow decay and the high initial SNR differently, the area under the curve is identical. Middle: The relation between SNR and Information, Eq.(5). Bottom: The Information versus pattern age calculated from the top and middle graph. The total information stored, equal to the area under the curve, is clearly larger when using small updates (thick curve) than when using large updates.

Using the Gaussian distribution of 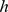 (denoted 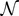) and the fact that the variances for lures and patterns become identical for small updates, the probabilities are calculated as a function of the Signal-to-Noise ratio. For instance, 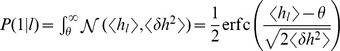. For the optimal threshold 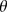, halfway between the Gaussians, one has for the probability of either mis-classification 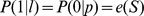, where the error rate equals 
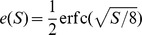
 with 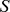 the signal-to-noise ratio, Eq.(1). The probability for correct responses is 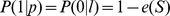. This yields the information as function of the SNR as
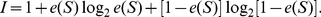
(5)This relation is plotted in Fig. 2(middle). If the output distributions totally overlap, the SNR is zero and the information is zero as well. For small SNR (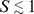), the information is linear in the SNR. Taylor expansion of Eq.(5) yields 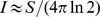. Importantly, the information is a saturating function of the SNR. For very high SNR, the two output distributions are almost perfectly separated, but because a pattern is either a learned pattern or a lure, there is maximally one bit of information per pattern. For example, doubling an already high SNR, only brings slightly more information.

The saturation has an important consequence when one wants to maximize information, illustrated in Fig. 2. The SNR decay is shown for two soft-bound learning settings, one with large updates, one with small updates. The total information is the sum of the information about all patterns; each pattern has a different age and hence SNR and information associated to it. Although the integral under the SNR curves is identical, the integral under the information curves, corresponding to the total information stored, is clearly smaller for the large updates. In other words, a high SNR wastes synaptic information capacity that otherwise could have been used to store more patterns. As an example, an initial SNR of 10 will achieve only 78% of maximal capacity (assuming exponential decay). The information capacity is maximized when this saturation is avoided and many patterns are stored with a low SNR, that is, when the synaptic updates are small.

This setup in principle requires a threshold precisely between the average output to pattern and lure, i.e. 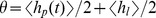. Hence the threshold should depend on the age of test pattern, but it would be difficult to imagine how this could be implemented. In the limit of small SNR, however, the information becomes independent of the precise threshold setting, and instead the threshold can be fixed, at say, 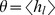.

### Soft and hard bound information capacity

**Figure 3:**
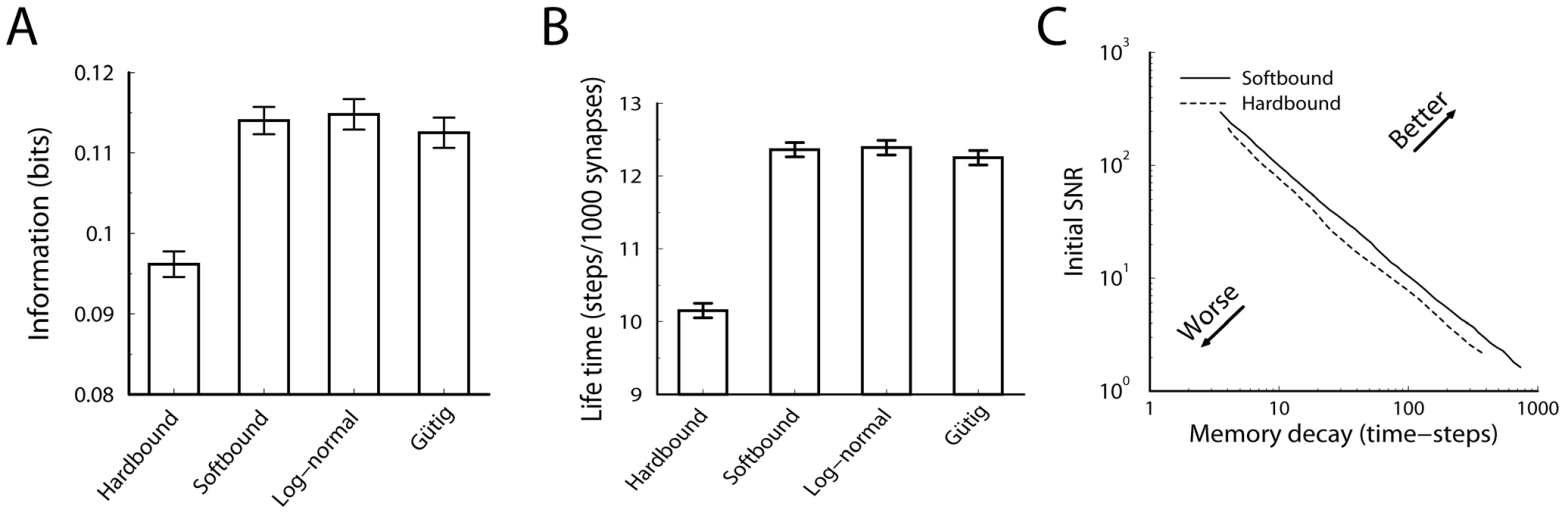
Comparison of hard-bound to soft-bound plasticity. A) The information capacity per synapse in the recognition task for a variety of plasticity rules. Up to numerical error, the soft-bound, log-normal and Gutig rule perform identically. B) Simulation of the lifetime of a memory for various plasticity rules. The lifetime was defined as the number of memories stored with a SNR above 30. Again soft-bound plasticity outperforms hard-bounds. C) The trade-off between memory decay time and initial memory strength for soft- and hard-bound plasticity. The amount of synaptic update was varied and the resulting fitted decay time-constant and the initial SNR was plotted. Ideally initial strength is high and memory decay time is long (top-right corner), but increasing one decreases the other. Soft-bound plasticity always leads to a superior trade-off.

Both soft-bound information capacity 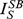 and hard-bound information capacity 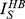 are calculated exactly in the limit of small updates in the Models section. We find
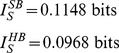
Thus the soft-bound plasticity can store more information. The improvement in performance is moderate though, some 18%. Fig. 3A shows the outcome of simulations that confirm these theoretical results, the soft-bound rule outperforms the hard-bound rule. These results raise the question whether other plasticity rules could increase capacity even further. That does not appear to be the case as we argue next.

### Alternative soft-bound plasticity rules

We simulated two additional soft-bound plasticity rules. The first comes from the empirical observation that the synaptic weight distribution can be fitted to a log-normal distribution [15]. Also the distribution of spine volumes, which correlates strongly with the synaptic strength, follows a log-normal distribution [16]. One way to obtain a log-normal distribution as a stationary weight distribution is to use an exponentiated Ornstein-Uhlenbeck process, where a decay term continuously pulls the weights back to the mean value [16]. Such a mechanism is difficult to reconcile with our setup. Instead we use that for small synaptic updates the soft-bound rule above yields a normal distribution. Exponentiation of the soft-bound plasticity rules yields
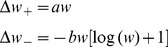
(6)For small updates this yields a log-normally distributed weight, with a mean equal to 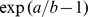.

The second rule is a polynomial plasticity rule [17], [33], which can be viewed as an interpolation between hard-and soft-bound.
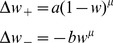
(7)If the exponent 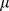 equals 1, one retrieves a soft-bound rule very similar to the soft-bound rule above (although not identical). The case 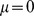 leads to Eq.(2) if hard bounds are imposed at 0 and 1. The performance of this rule improves gradually from hard-bound and soft-bound as 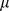 increases from 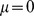 to 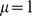, interpolating from the hard- to soft-bound case. To examine if this rule can outperform the earlier soft-bound rule, we choose a value outside this range, 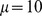.

Both the log-normal and polynomial rule perform as well as the original soft-bound rule, but neither did better, Fig. 3A. In the Model section we show that a large class of soft-bound rules indeed have the same capacity and that this does not depend on the precise parameter values, as long as the updates are small. To further corroborate the optimality of soft-bound plasticity, we numerically optimized plasticity rules for which both potentiation and depression are general second order polynomials in 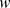


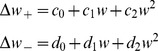
(8)To examine if the soft-bound plasticity could be outperformed, the coefficients 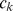 and 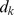 were varied to find numerically their best values. For numerical performance, the weight wa discretized in 200 bins and the number of synapses was limited to 100 so that there was no saturation from discretization [25]. The information capacity was identical to the soft-bound rules, but no improvement could be achieved by allowing these polynomial update rules. Finally, the result matches the maximum information capacity of discrete synapses for which a more exhaustive optimization is possible [25]. Together these results suggest that for this class of learning rules, soft-bound plasticity performs optimally.

### Memory lifetime

Although the use of small synaptic updates maximizes information capacity, in practice there are issues with using small updates. First, the low SNR of each memory renders the pattern/lure discrimination sensitive to noise, such as noise from synaptic variability or other inputs to the neuron. Secondly, in recurrent networks, such as the Hopfield network, errors made by a single neuron can be amplified by the network. Moreover, experimental evidence suggests that synaptic plasticity protocols can induce substantial changes in a synapse. Finally, soft-bound plasticity with small updates leads to narrow weight distributions (i.e. with a small variance), while the observed synaptic weight distributions are relatively broad, consistent with larger updates.

We therefore define a second storage measure to compare hard and soft-bounds. We use the same single neuron paradigm used above but define the memory lifetime as the number of recent patterns that the neuron stores with SNR above a predefined threshold [34]. Similar measures have been defined for networks [35]. For instance, in the Hopfield network, the single neuron error rate should stay below 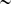
0.36% to prevent an avalanche of errors in the recurrent activity [1]. This error rate corresponds to a SNR of about 30, and corresponds to a regime where the synaptic updates are no longer small. A full analysis of capacity of recurrent networks is rather more involved [36], [37], but the current approach is sufficient for our purposes.

Because the SNR decays exponentially with soft-bound plasticity (Models), 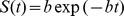, the number of memories stored above threshold is easily calculated. To find the maximum lifetime the plasticity parameters have to be optimized, as with too small updates the SNR might never become high enough, while too large updates would lead to rapid over-writing. One finds the optimal 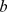 to be 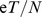, leading to a life-time
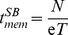
where 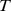 is the imposed threshold and 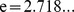 is Euler's number. The memory lifetime increases linearly with the number of synapses and decreases with SNR threshold.

The lifetime in the hard-bound case can be approximated by taking only the lowest order term in the expression for the SNR decay (see Models), yielding
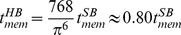
Thus again soft-bound plasticity is superior to hard-bound plasticity, on this measure by some 20%.

The theory is confirmed by the simulations in which we numerically maximized the lifetime by changing the synaptic update. Too small updates would lead to none or few patterns above the threshold, while too large updates speed up the decay. The lifetime is plotted normalized by the number of synapses. The soft-bound plasticity, as well as the other soft-bound variants, outperform hard-bound, Fig. 3B.

As indicated, the SNR decay leads to a trade-off between initial SNR and memory decay time. The current performance measure sets a somewhat arbitrary threshold to resolve this. However, the result is independent of the precise threshold setting. Fig. 3C shows the initial SNR versus the forgetting time, as we parametrically vary the size of the synaptic update. For both hard and soft-bound plasticity, SNR and decay time are inversely proportional over a large range. The soft-bound plasticity is superior, as it always gives a longer lifetime for the same initial SNR, no matter what the preferred trade-off between memory strength and retention time. Note that the information capacity analyzed in the previous section is reflected in Fig. 3C as well. Apart from a constant, the information capacity approximately equals the product of decay-time and initial SNR in the limit of small SNR, i.e. in the right region of the graph.

### Feed-forward inhibition and sparse codes

In the simulations and the analysis we made two assumptions. First, the input patterns take positive and negative values, but in biological systems it would seem more natural to assume that the inputs are spikes, that is, 1s and 0s. Secondly, we tacitly used fixed feedforward inhibition to balance the excitatory inputs and ensure that the effective synaptic weight has zero mean. Without these assumptions the SNR, and hence the information capacity, for both hard- and soft-bound plasticity decreases significantly.

The reason for the decreased SNR is easiest seen by analyzing the variance in the output in response to a lure pattern. Because a lure pattern will be completely uncorrelated to the weights, the variance can be written as
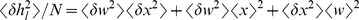
(9)The higher this quantity, the smaller the SNR, the worse the performance of both the information measure and the memory lifetime. We now examine the second and third term in detail.

**Figure 4:**
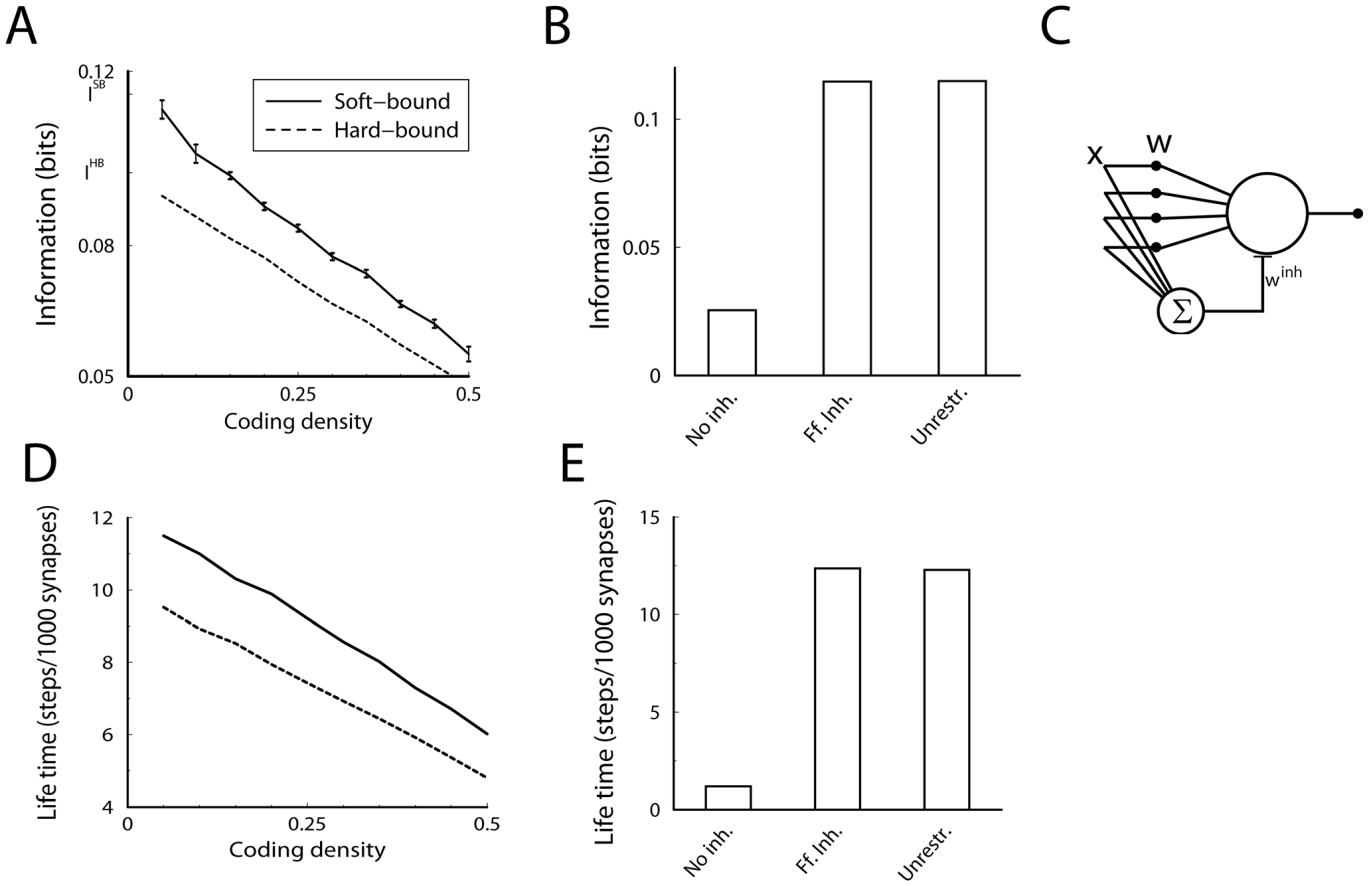
Effect of coding density and inhibition on performance. A. The synaptic information capacity versus the coding density for soft-bound (solid line) and hard-bound (dashed line) plasticity, when taking 0s and 1s as inputs. When coding density is 1/2, the capacity is approximately half of what it is when using 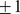 as inputs. However, low coding density improves the synaptic information capacity and in the limit of very sparse codes (utmost left in the graph) the capacity reaches that of Figure 3A. B. Effect of feed-forward inhibition on capacity under soft-bound plasticity. Using excitatory synapses (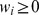) without inhibition, capacity is strongly reduced (‘No inhibition’). Adding feed-forward inhibition maximizes information capacity (‘Feedforward inhibition’). Equivalently, high capacity is achieved when the plasticity rules are defined such as to allow for negative weights (‘Unrestricted’). C. Possible circuit to implement feed-forward inhibition. D+E. Effects of coding density and inhibition on the information (panel A+B) on the information are mirrored by the effects on the memory lifetime.

In the above results the second term in Eq.(9) was zero, because we used 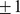 inputs with a coding density of 1/2 (equal probability of high and low input) so that mean input 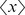 was zero. When using 0/1 inputs this term is no longer zero. This strongly reduces the capacity at high coding density, Fig. 4A. However, when coding is sparse so that most inputs are zero and only a small fraction are one, the mean input 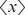 is again close to zero and the information capacity approaches the theoretical maximum, Fig. 4A. This is also the case for the memory lifetime, Fig. 4D. Note that in simulations with sparse codes, we scale the plasticity such that the balance between depression and potentiation is maintained. For instance, for the hard-bound plasticity, we use 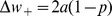, 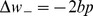, where 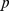 denotes the probability for a high input. Not doing so, would decrease capacity.

The third term in Eq. (9) is proportional to the mean weight 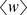 and reflects changes in the output due to changes in the number of active inputs. When only excitatory inputs are used and hence the mean weight is non-zero, the capacity is reduced, Fig. 4B. In the simulations zero mean weight was effectively achieved by implementing feed-forward inhibition. Hereto we introduced an inhibitory partner neuron that receives the same inputs as the original neuron and that calculates the un-weighted sum 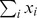. This neuron then inhibits the output neuron with an inhibitory weight 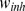, Fig. 4C. The total output of the neuron is 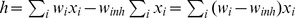. If the inhibitory weight is adjusted to balance the mean excitatory weight (
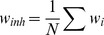
), optimal capacity is obtained, Fig. 4B. Similar arguments have been made in a variety of memory models [38]–[40].

In this idealized setup, feed-forward inhibition is mathematically equivalent to making the mean weight zero by allowing negative weights and adjusting the plasticity rules. For instance, for the hard-bound plasticity, this is achieved by just moving the lower weights bound to −1; for soft-bound plasticity, the depression rule is redefined to 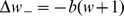. Indeed, the information per synapse is identical, Fig. 4B. This same argument applies again to the memory lifetime and thus it behaves in parallel, Fig. 4E.

### Imbalanced potentiation and depression

**Figure 5:**
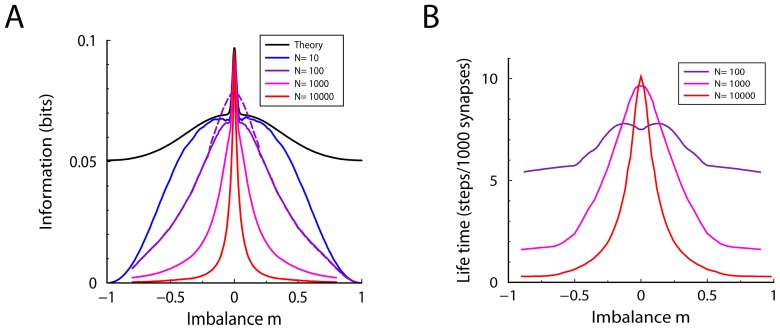
The effect of imbalance between potentiation and depression on the capacity measures for hard-bound plasticity. A. The Information capacity showing the theory (black) as well as simulations for 10, 100, 1000, and 10000 synapses for 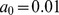 (blue, violet and magenta). In contrast to the balanced case (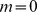) the capacity depends on the number of synapses. A larger synaptic update always decreases capacity in the balanced case, but can improve capacity in the imbalanced case (dashed curve, 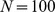, 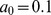). B. Memory lifetime decreases when potentiation and depression are imbalanced. The memory lifetime was optimized w.r.t 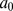 for every setting of 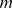 and 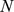.

A further potential problem with hard-bound plasticity is that highest capacity is only attained when potentiation and depression are exactly balanced [23]. In contrast imbalance in soft-bound plasticity shift the mean weight but do not affect the capacity or the lifetime. Our theory allows for an exact analysis of imbalance (see Models). We set the potentiation strength to 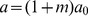 and the depression to 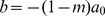, so that 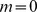 recovers the balanced case. Capacity is indeed diminished if the amount of the potentiation event does not exactly balance the depression event. Simulation and theory are shown in Fig. 5A and reveal an interesting stupa-like shape: The information is maximal in the balanced case (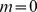). For small imbalances favouring either LTD or LTP, the information decreases rapidly, following the theory (black curve, see Models). However, for larger imbalance the decrease in information is moderate. In this region, the weight distribution approaches a narrow exponential. The fast decay of the signal caused by the imbalance is counter-acted by the reduction of the variance of the weight distribution as 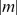 increases. Due to this effect, the initial SNR increases but the information saturates unavoidably, even for small updates. This is reflected in the fact that the simulations deviate from the theory and show progressively less information per synapse as the total number of synapses of the neuron increases.

The width of the peak around 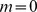 is given by the condition 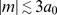. Thus the peak can be widened by increasing the event size 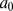 (an example is shown with the dashed line). However, a larger event size also increases saturation when 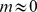. Thus unlike the balanced case, where a small update always maximized mutual information, the optimal synaptic update in the imbalanced case is dependent on the imbalance parameter and the number of synapses. In parallel, the memory lifetime decreases when potentiation and depression are imbalanced, Fig. 5B.

## Discussion

We have studied plasticity rules that include the experimental observation that plasticity depends on the synaptic weight itself. Namely, the relative amount of potentiation decreases as the synapse gets stronger, while the relative amount of depression shows no such dependence. This means that the synaptic weight is automatically bounded. Using an information theoretic framework we have compared these plasticity rules to hard-bound plasticity in an online learning setting. We found that the information storage is higher for soft-bound plasticity rules. Contrasting the prototypical soft-bound and hard-bound plasticity rules, the improvement can be calculated analytically. In addition, a wide class of soft-bound plasticity rules lead to the same increased capacity and we suggest that soft-bound rules in fact maximize the synaptic capacity. Furthermore, we examined an alternative capacity measure that determines how many patterns can be stored above a certain threshold. This memory lifetime measure also improved for soft-bound plasticity.

The improvement in performance is moderate for both capacity measures, some 18%. However, it should be stressed that *a priori* there was no reason to assume that weight dependence would actually improve capacity, it could have led to a lower capacity. Moreover, naively one might have guessed, errorneously, that the uniform weight distribution associated with hard-bound plasticity would be optimal. The difference in capacity means that for hard-bound plasticity to perform as well as the soft-bound one, 18% more synapses would be required. This is a significant space and metabolic cost for the nervous system as synapses consume about 60% of the brain's energy [41].

Given that the difference between hard and soft-bound is only quantitative, it is difficult to point to the cause for the difference, besides our mathematical derivation. Our intuition is that the hard-bounds lead to a deformation of the weight distribution. As a result, the decay back to equilibrium is a sum of exponentials. This means that the decay is always somewhat faster than for the soft-bound case for identical initial SNR, Fig. 1c.

Our study reveals the following criteria to optimize synaptic storage capacity: 1) use soft-bound rules, 2) ensure that the mean input is close to zero, for instance by using sparse codes, 3) ensure that the mean weight is close to zero, for instance by implementing balanced feed-forward inhibition, and finally, if one wants to maximize the mutual information, 4) update the synapse in small amounts.

There have recently been a number of studies on information storage and forgetting in synapses with a discrete number of states. The continuous synapses studied here have an equal or superior capacity, as they can always effectively act as discrete ones. An earlier study of discrete synapses argued that balanced hard-bound rules are superior to soft-bound rules when long memory lifetime is required [23]. These concerns don't apply to continuous synapses, and we believe that our performance measure based on Shannon information is a more fundamental one. That said, the precise biological objectives and constraints for synaptic plasticity are unknown. Furthermore it should be noted that, as for any optimization argument, another criterion would likely yield a different optimal rule. For instance, one-shot learning would require large updates and thus yields low information, while sensitivity to input correlations has been shown to require a rule intrapolating between hard and soft-bound [17].

The results do not depend on the precise form of the soft-bound plasticity rules. Biophysically, numerous mechanisms with any kind of saturation, could lead to soft-bound plasticity. At the level of a single synapse one could even argue that weight-*independent* plasticity would be difficult to achieve bio-physically, as many biophysical signals such as Ca influx and AMPA insertion are likely affected by the synaptic weight itself. Interestingly, the spine volume grows substantially as the synapse undergoes potentiation [42]. At first glance this suggests that the spine readies itself for potential further strengthening, but it has been suggested that actually the increase in spine volume reduces the calcium transients, limiting further potentiation, and therefore giving rise to soft-bound plasticity [43], [44].

Apart from the increased capacity, weight dependence has other important consequences for plasticity. First, the weight dependence leads to central weight distributions, consistent with data, both measured electro-physiologically and microscopically. Second, competition between synapses is weaker for soft-bound rules because depressed synapses never completely disappear. Finally, in soft-bound plasticity the mean weight remains sensitive to correlations [19], in line with recent evidence in [20].

In an earlier study we reported that soft-bound STDP plasticity leads to shorter retention times than hard-bound plasticity in both single neurons and networks [21]. In that study the update size was not optimized as done here, but instead the average synaptic update per pre-post spike pairing was set the same for hard-bound and soft-bound plasticity. Fully consistent with the results in the Models section, for the same update soft-bound plasticity leads to quicker decay (proportional to the update size) than hard-bound (proportional to the update size squared) [23]. Because of the difference in the setup, the SNR measure used there can not directly be compared with the one derived here. Characterization of the information storage was not carried out in that setting, but we see no reason why the current results would not hold for STDP learning.

Soft-bound plasticity is certainly not the only way to prevent run-away plasticity. Apart from hard-bounds, BCM theory [45], and normalization models [11] are some of the best known alternatives. Soft-bound plasticity however provides one of the easiest solutions to run away plasticity, as it does not require a running average of activity (needed for BCM) or knowledge of the other synapses onto the neuron (as needed in normalization models). Yet, soft-bound plasticity can co-exist with those mechanisms, as well as with homeostatic processes, and thus can be part of a larger set of mechanisms to keep neural activity and plasticity in check. This study suggests that this can be done without losing any storage capacity, but instead gaining some.

## Models

### Calculation of information capacity

In this section we calculate the capacity of soft and hard-bound plasticity analytically. To calculate the capacity we concentrate on one single synapse, as the Signal-to-Noise scales linearly with the number of synapses. We artificially distinguish the pattern that is to be learned from all the other patterns that are presented subsequently and erase the memory of this pattern, although of course, no pattern stands above the others; the same plasticity rules underlie both the storage and the forgetting processes. Throughout we assume that the synaptic updates are small. This prevents saturation in the relation between SNR and Information, Fig. 2B, ensuring maximal information.

To calculate the information storage we need to study how the synaptic weight decays after learning. As the weight updates are small, a Fokker-Planck equation for the weight distribution describes the decay of the synapse as it is subject to the learning of other patterns. The synaptic weight distribution 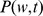 evolves as [13]

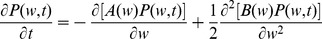
where the drift is 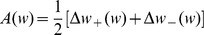, the diffusion term is 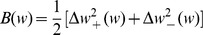, and where 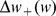 and 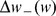 denote the weight change associated with potentiation and depression. We denote an average over many trials with angular brackets, the variance is denoted 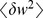, and the equilibrium mean weight is denoted 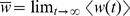.

### Soft-bound plasticity rule

We first calculate the information capacity for the soft-bound rule. The solution Fokker-Planck is complicated, but when the jumps are much smaller than the standard deviation of the distribution, that is, 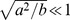, the diffusion term is approximately constant 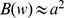. In this limit, the equilibrium weight distribution, defined by 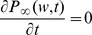, becomes a narrow Gaussian distribution 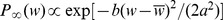, with mean 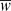 and variance 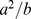. Note, that validity of the Fokker-Planck approximation itself only requires the weaker condition that the updates themselves are small, 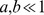.

We consider what happens to a given synapse when a pattern is learned. Suppose that at time zero, the synapse gets a high input and is thus potentiated (the calculation in the case of depression is analogous). Right after the potentiation event, the weight distribution is displaced with an amount 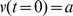, where 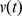 denotes the displacement of the weight. For small updates one has 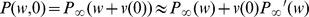.

**Figure 6:**
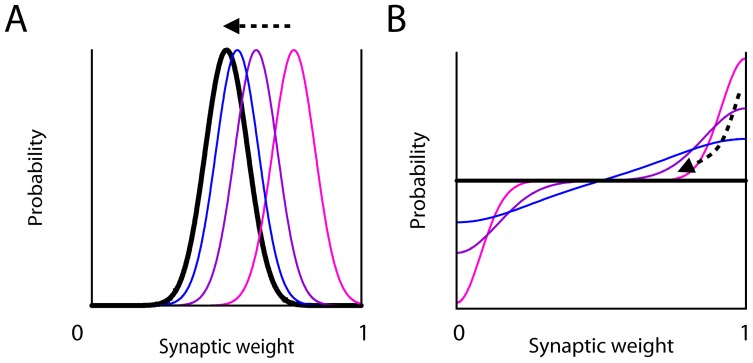
Illustration of decay of the weight distributions after a potentiation event. The distribution right after the potentiation is shown by the magenta curve; as time progresses (indicated by the arrow) it decays back to the equilibrium distribution (thick black curve). A) With soft-bound plasticity the distribution is displaced but maintains its shape. During the overwriting it shifts back to the equilibrium distribution. B) With hard-bound plasticity, the distribution distorts after the potentiation due to the presence of the bounds. As it decays back to the equilibrium this distortion flattens out.

After this event, the synaptic weight is subject to random potentiation and depression events caused by the learning of patterns presented subsequently. During this the perturbation of the synaptic weight distribution will decay, until finally the distribution equals the equilibrium again and the potentiation event will have lost its trace. The perturbed distribution can be plugged into the Fokker-Planck equation to study its decay back to equilibrium. Because 
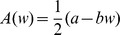
 is linear in 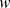 and 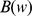 is approximately constant, it can be shown that the probability obeys
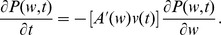
This is a transport equation, which means that during the overwriting of the synapse by the subsequent patterns the distribution shifts back to its equilibrium value, but maintains its shape, Fig. 6A. From this equation it follows that the mean weight obeys 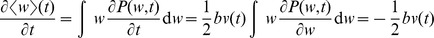. The mean weight decays back exponentially with a time-constant 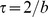 as 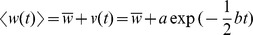.

The output signal is found by probing the synapse with a ‘1’ (high input). Assuming perfectly tuned feed-forward inhibition, the mean output signal is 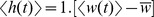. As the variance of the weight distribution and hence the variance of the output is to first approximation not affected by the plasticity, the signal-to-noise is 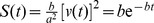. The information follows as
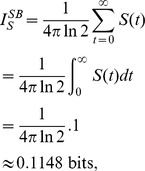
which matches simulations.

In the simulations we found the same information for other soft-bound plasticity rules. For small updates, a Taylor expansion of the drift 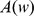 can always be made, yielding the linear term in the drift, furthermore the diffusion term becomes independent of 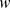 close enough to the center of the distribution. These approximations become perfect in the limit of small update updates. Therefore most soft-bound rules can be mapped to the above one, yield a narrow Gaussian as equilibrium distribution and have the same capacity. Finally, one can construct soft-bound rules in which the linear term in the drift is absent [23]. However, also for those cases we numerically found the same capacity.

### Calculation of information capacity hard-bound rule

We repeat the calculation for hard-bound plasticity. We impose hard-bounds at 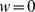 and 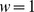, that prevent the weights from crossing minimal and maximal values. This corresponds to imposing the boundary conditions 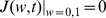 to the Fokker-Planck equation, where 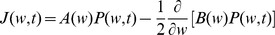 is the probability flux. As shown below, the capacity is optimal when potentiation and depression are matched. In this case, 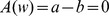 and 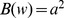. The equilibrium distribution is the uniform distribution 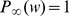, with mean weight 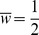. The boundary conditions simplify to 
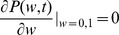
.

At time 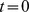 the synapse is potentiated with a small amount 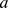. The average weight increases by an amount 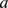. The weight distribution, which expresses the probability to find the weight of a certain value, is displaced, creating a bump at the upper boundary and a dip at the lower boundary, Fig. 6B. The distribution becomes 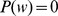 for 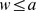, 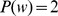 for 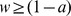, and 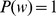 otherwise. We approximate this perturbation as 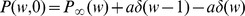, where 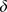 is the Dirac-delta distribution.

As above, the synaptic weight is subject to random potentiation and depression events caused by the learning of patterns presented subsequently. Solving the Fokker-Planck equation gives that the perturbation of the synaptic weight distribution decays as
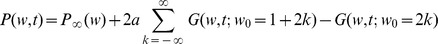
where the Green's function 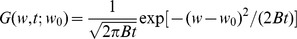 is the solution to the diffusion equation in infinite space when all weights are initially concentrated at 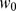. The sum over 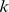 represents the ‘mirror charges’, needed to satisfy the boundary conditions, preventing the synaptic weight escaping from the interval between 0 and 1; similar equations arise in neural cable theory, e.g. [46].

The derivative of the mean weight follows from the Fokker-Planck equation after integration by parts as
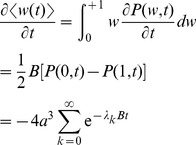
where 
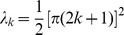
. Integration gives that the weight decays as 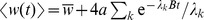. Note that the inverse of the slowest time-constant 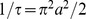 is proportional to the update squared, in contrast to the soft-bound case were it is linearly proportional to the synaptic update [23].

The variance in the output equals that of the uniform distribution and is 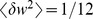. Thus, assuming perfect feed-forward inhibition, the signal-to-noise ratio is 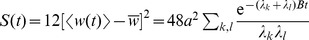. Hence for small plasticity events the information is
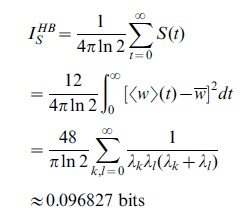
where the sum was calculated numerically. For practical purposes the sums over 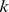 and 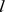 can be truncated above 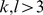 as the contributions to the sum rapidly diminish.

### Imbalanced plasticity

For the soft-bound plasticity, the ratio between potentiation and depression determines the mean synaptic weight, but the capacity does not depend on it. For hard-bound plasticity, however, the situation is more complicated. Imbalance between potentiation and depression leads to a deformation of the steady state distribution and speeds up the decay after perturbations, changing the capacity.

We assume that the size of the potentiation event is 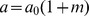, and depression 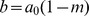, where 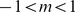 parameterizes the imbalance. To calculate the capacity one again needs to calculate the reaction to a perturbation (potentiation or depression) away from the equilibrium. But as now 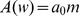 is no longer zero, the mirror charges trick does not work. Instead using standard Sturm-Liouville theory, the solution to the Fokker-Planck with boundary conditions 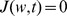 can be written as a series expansion
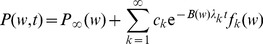
(10)where the steady state distribution is 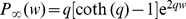 and where 
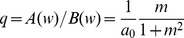
 is the re-scaled drift. The eigenfunctions 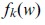 are
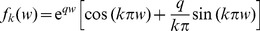
with eigenvalues 
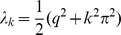
. The imbalance, expressed by the factor 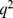, speeds up the decay to equilibrium. The coefficients 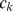 in Eq.10 follow from the initial condition. As above we first consider a single potentiation event of size 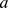. This will shift the weight distribution as 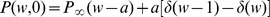; the coefficients 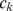 (normalized to 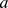) follow as
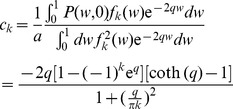
From this we calculate the evolution of the mean weight 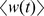 using Eq.10. Each eigenfunction contributes an amount 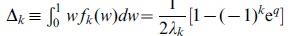 to the perturbation of the mean weight. Averaging potentiation and depression events, the Information becomes
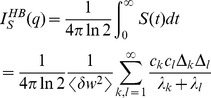
This expression is plotted in Fig. 5A (black line) as a function of 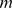.

### Computer simulations

The computer code is made available on the first author's website. To examine the various plasticity rules, we presented typically one million patterns to the neuron, one pattern at every time-step. Every pattern led to an update of the synaptic weights according to the plasticity rules. After each time-step, the output of the neuron in response to this pattern, to its predecessors, as well as to lures was measured. The mean and the variance of the neuron's output were collected online as a function of the pattern's age, that is how many time-steps ago the pattern was presented. From this the SNR and information was calculated at the end of the simulation using Eq.(5) and (1).

Importantly, unlike the analysis above, the simulations are not restricted to small synaptic updates. Furthermore, the Gaussian assumption can be dropped in the simulations. In that case all the neuron's responses were stored, and the information was calculated at the end of the simulation using the full response distributions to patterns and lures. We found results were the virtually identical. It required, however, much more computer memory and time than the first method.

Unless indicated otherwise, for the figures we used 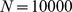 synapses for the lifetime calculations. For efficiency reasons we used 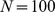 to calculate the information measure as the scaling with the number of synapses is trivial.
